# Intramedullary nailing versus external ring fixation for tibial shaft fractures: an explorative analysis of muscle strength from the IMVEX trial

**DOI:** 10.1007/s00402-025-05995-6

**Published:** 2025-07-18

**Authors:** Peter Larsen, Rasmus Stokholm, Jan Duedal Rölfing, Juozas Petruskevicius, Morten Kjerri Rasmussen, Steffen Skov Jensen, Rasmus Elsoe

**Affiliations:** 1 Aalborg University Hospital, Aalborg, Denmark; 2 Aarhus University Hospital, Aarhus, Denmark; 3 Regional Hospital Central Jutland, Viborg, Denmark

**Keywords:** Tibial shaft fracture, Muscle strength, Recovery, Physical outcome

## Abstract

**Background:**

To explore the effect of intramedullary nailing versus external ring fixation for patients with tibial shaft fractures on 12-month maximal isometric muscle strength for knee extension and knee flexion. We hypothesise that patients treated with external ring fixation will show significantly better maximal isometric muscle strength for knee extension and knee flexion 12 months after surgery compared to treatment with intramedullary nailing. Moreover, we aim to explore the association between muscle strength and the Knee Injury and Osteoarthritis Outcome Score (KOOS) at 12 months following surgery.

**Methods:**

A pragmatic multicentre randomised, non-blinded trial with a two-group parallel design. This study is an explorative analysis of the IntraMedullary nailing Versus EXternal ring fixation Trial (IMVEX). 67 patients with a tibial shaft fracture were randomized to external ring fixation or intramedullary nailing. The primary outcome: maximal isometric muscle strength for knee extension and knee flexion. Secondary outcome was KOOS.

**Results:**

No statistically significant difference in muscle strength between treatment with intramedullary nailing and external ring fixation was observed (*P* < 0.31). Examination of maximal isometric muscle strength showed significant difference between the injured and the non-injured leg (knee extension: mean difference of 53N, (95%CI 33.9–72.4), knee flexion: mean difference of 35N, (95%CI 21.2–47.9)) The association between KOOS subscale scores and the relative difference in muscle strength for knee extension showed significant and moderate to high correlation (0.4–0.8) for all subscales.

**Conclusion:**

Muscle strength of knee flexion and extension was markedly decreased one year after fracture. We observed no statistically significant difference in recovery of muscle strength between treatment with intramedullary nailing and external ring fixation. Results indicate that a decrease in muscle strength is associated with worse patient-reported outcomes for all five KOOS subscales.

**Trial registration:**

NCT03945669.

**Level of evidence:**

II.

## Introduction

Tibial shaft fractures are common injuries with an incidence of 16.9/100,000/year [1]. A common treatment method is intramedullary nailing by an infra- or supra-patellar approach [2]. Tibial shaft fractures are associated with a considerable complication rate and risk of musculoskeletal complaints such as knee pain, limitations in activities of daily living and restrictions in quality of life (QOL) [3–10].

Muscle strength of the lower extremities is well known to be associated with independence, ambulation and one-year mortality following long-bone fractures in the elderly [11, 12]. Standing from a chair, climbing stairs, jumping, running and participating in recreational activities are important physical tasks associated with muscle strength of the lower extremities, regardless of age [13–16]. A few studies have reported on muscle strength following the treatment of tibial shaft fractures; all reporting restrictions in muscle strength during the first years following fracture [6, 7, 17–21]. Most studies reporting of muscle strength following tibial shaft fractures included solely patients treated with intramedullary nailing from the infrapatellar approach, however the suprapatellar approach for intramedullary nailing has become increasingly popular and is reported with better functional outcomes [7, 17–20, 22].

Theoretically, treatment with an external ring fixator may have an advantage regarding muscle strength compared to treatment with intramedullary nailing, as the surgical procedure is minimally invasive and does not necessitate surgery through the knee joint for nail insertion [23]. Contradictory, a period of 3–6 months with an external ring fixator may influence patients’ physical activity level compared to patients treated by intramedullary nailing. Previously, three randomised controlled trials have investigated the effect on patient-reported outcomes and pain from treatment with intramedullary nailing versus external ring fixation for tibial shaft fractures [24–26]. Objective data for muscle strength are not available from these studies. None of the studies reported a significant difference in the primary outcome.

The aim of this study was to explore the effect of intramedullary nailing versus external ring fixation for adult patients with tibial shaft fractures (AO/OTA fracture types: AO-42-) on 12-month maximal isometric muscle strength for knee extension and knee flexion. Moreover, we aim to explore the association between muscle strength and the Knee Injury and Osteoarthritis Outcome Score (KOOS) measured at 12 months following surgery.

We hypothesise that patients treated with external ring fixation will show significantly better maximal isometric muscle strength for knee extension and knee flexion 12 months after surgery compared to treatment with intramedullary nailing.

## Methods

### Study design

This study is an explorative analysis of the IntraMedullary nailing Versus EXternal ring fixation Trial (IMVEX) comparing intramedullary nailing versus external ring fixation for adult patients with tibial shaft fractures [26]. Details of the study design have previously been published [23]. In brief, the IMVEX study was a pragmatic multicentre randomised, non-blinded trial with a two-group parallel design including 67 patients with a tibial shaft fracture randomised to treatment with either external ring fixation or intramedullary nailing. The IMVEX trial compare 12-months patient-reported outcome (KOOS) and indicated an advantage for external ring fixation; however, it lacked power, i.e., potentially masking for a clinically relevant difference [26].

The published study protocol provides a detailed description of the two surgical procedures (study interventions) [23]. Notably, the use of either the supra- or infrapatellar approach for intramedullary nailing was at the discretion of the operating surgeon.

The study was pre-registered at ClinicalTrials.gov (*NCT03945669*) and has been approved by the Ethics Committee of the North Denmark Region (N-20180075) and the Danish Data Protection Agency (ID 2019-151). The trial's reporting follows the Consort guidelines [27].

### Patients

Patients were enrolled from six hospitals in northern Denmark: Aalborg University Hospital, Aarhus University Hospital, and regional hospitals in Hjoerring, Thisted, Farsoe, and Viborg.

We included adult patients (≥ 18 years of age) with an acute tibial shaft fracture, deemed operable with IMN by the attending senior orthopaedic trauma surgeon, who were willing to be randomised and provided oral and written informed consent.

Excluded were patients with:

- Open fractures excluding the use of intramedullary nailing.
- History of severe systemic diseases or cancer.
- Pregnancy.
- Patients without gait function before fracture.
- Non-Danish citizens.
- Patients living outside the hospital treatment area.
- Patients with major cognitive impairment.

### Outcome assessment

#### Primary outcome and endpoint

The primary outcome for this explorative analysis was maximal isometric muscle strength for knee extension and knee flexion at 12-month follow-up measured by a strap-mounted dynamometer attached to the wall (Mecmesin AFG2500, Mecmesin Ltd, West Sussex, UK). Muscle testing was performed with the patient sitting on an examination table. Knee extension strength was tested at 60° flexion of the knee, and knee flexion strength was tested at 90° flexion of the knee. A 30-s pause was maintained between all tests. The test was repeated three times for both legs, and the highest value was used for the analysis. The test set-up was described and validated by Rathleff et al. [28]. To minimize the risk of a re-fracture patients reporting pain from the fracture area at manual load test were excluded from participating in the maximal isometric muscle test.

#### Secondary outcome

To analyse the association between maximal muscle strength and patient-reported function and quality of life, we included the Knee Injury and Osteoarthritis Outcome Score (KOOS) [29]. The KOOS is a validated instrument to evaluate patients’ opinion of their knee and associated problems [29]. The questionnaire includes five subscales: pain, symptoms, ADL, sport/rec and QOL [29]. A total score of 100 indicates no symptoms, and 0 indicates major symptoms [29]. The KOOS questionnaire has previously been used in several studies investigating the outcome of tibial shaft fractures [6, 7, 25, 30]. The KOOS was completed at the 12-months follow-up a the orthopaedic outpatient clinic.

### Statistics

The between-group difference in muscle strength was reported in Newton (N) for the injured and non-injured leg and as a relative difference between legs (%) to normalise the nature of muscle strength between young and older patients and between genders in the analyses.

A paired t-test was used to compare the maximal isometric muscle strength between the injured and the non-injured leg for knee flexion and extension. A paired *t*-test was used to compare the relative difference in maximal isometric muscle strength between intramedullary nailing and external ring fixation for knee flexion and extension.

Pearson’s correlation was used to explore the association between KOOS subscale scores and the relative difference in muscle strength for knee flexion and extension.

Pearson’s correlation was interpreted as 0–0.3 low, 0.4–0.6 moderate and 0.6–1.0 high [31].

## Results

In the IMVEX trial, a total of 67 patients were randomised to either intramedullary nailing (*N* = 33) or external ring fixation (*N* = 34). The 12-month follow-up was completed by 31 patients assigned to intramedullary nailing and 34 patients assigned to external ring fixation.

Data for muscle strength were available for 24 patients assigned to intramedullary nailing and 29 patients assigned to external ring fixation. Baseline characteristics of 53 patients available for muscle strength analysis are presented in Table 1.

**Table 1:** Patients baseline charrecteristics precented by treatment group

	Intramedullary nailing	External ring fixation
Number of patients with muscle strength data, *n*	24	29
Age at time of fracture, mean (range)	47.0 (17.6)	44.3(17.9)
Sex, *n* (%) female	8 (33%)	10 (34%)
Height cm, mean (SD)	177.7 (10.6)	176.7 (9.9)
Mass kg, mean (SD)	83.3 (16.2)	76.7 (17.0)
Body mass index kg/m^2^, mean (SD)	26.4 (17.6)	24.5 (4.6)
RF weeks in frame, mean (SD)	NA	23.6 (9.3)
IMN entry, *n* (%)		
Infrapatellar	6 (25%)	NA
Suprapatellar	18 (75%)	NA
AO/OTA fracture classification, *n* (%)		
A	13 (54%)	20 (69%)
B	10 (42%)	7 (24%)
C	1 (4%)	2 (7%)

*RF* external ring fixation; *IMN* intramedullary nailing; *SD* standard deviation; *n* number

### Muscle strength intramedullary nailing versus external ring fixation

Differences in knee extension and knee flexion muscle strength between patients treated with intramedullary nailing and external ring fixation showed no significant difference between groups, *P* > 0.31. Comparable results were observed when comparing the relative difference in muscle strength between treatment groups, *P* > 0.14. (Tables 2, 3).

**Table 2:** Muscle strength overall and by group

	Overall (*N* = 53)	Intramedullary nailing (*N* = 24)	External ring fixation (*N* = 29)
Knee flexion			
Injured, *N* (SD)	213.5 (116.8)	198.6 (111.8)	225.9 (121.4)
Non-injured, *N* (SD)	248.1 (113.8)	242.2 (115.3)	252.9 (114.3)
Mean difference injured/non-injured, *N*(*p*)	34.5 (0.001)	43.7 (0.001)	26.9 (0.003)
Knee extension			
Injured, *N* (SD)	285.6 (131.1)	282.9 (142.6)	287.9 (123.3)
Non-injured, *N* (SD)	338.7 (132.5)	359.2 (142.6)	321.8 (110.9)
Mean difference injured/non-injured, *N*(*p*)	53.1 (0.001)	76.3 (0.001)	34.0 (0.003)

**Table 3:** Muscle strength—difference between intramedullary nailing (IMN) and external ring fixation (RF)

	Knee flexion	Knee extension
Mean difference between IMN/RF injured, *N*(*p*)	27.3 (0.40)	5.0 (0.89)
Mean difference between IMN/RF non-injured, *N*(*p*)	10.6(0.74)	− 37.3(0.31)
Relative difference between IMN/RF(%) (*p*)	4.9 (0.35)	8.1(0.14)

### Muscle strength injured versus non-injured leg

Examination of maximal isometric muscle strength for knee extension showed a significant difference between the injured and the non-injured leg (mean difference of 53N, (95%CI 33.9–72.4), *P* = 0.001). The relative difference between the injured and the non-injured leg for knee extension was 15.4% (95%CI 10.0–20.8).

Examination of maximal isometric knee flexion strength showed a significant difference between the injured and non-injured leg (mean difference of 35N, (95%CI 21.2–47.9), *P* = 0.001). The relative difference between the injured and the non-injured leg for knee flexion was 14.7% (95%CI 9.5–19.9) (Table 3).

### Associations between KOOS subscale scores and relative difference in muscle strength

The association between KOOS subscale scores and the relative difference in muscle strength for knee extension showed significant and moderate to high correlation (0.4–0.8) for all subscales (Table 4). Results indicated that lower KOOS scores were associated with increasing knee extension muscle strength differences between the injured and non-injured leg. Comparable results were observed for both the intramedullary nailing and external ring fixation groups (Table 4).

**Table 4:** Association between KOOS subscale scores and the relative difference in muscle strength

KOOS	Knee extension						Knee flexion					
	Overall (*N* = 53)		Intramedullary nailing (*N* = 24)		External ring fixation (*N* = 29)		Overall (*N* = 53)		External ring fixation (*N* = 29)		Intramedullary nailing (*N* = 24)	
	*r*	*P*	*r*	*P*	*r*	*P*	*r*	*P*	*r*	*P*	*r*	*P*
Pain	0.6	0.001	0.7	0.001	0.5	0.03	0.5	0.001	0.4	0.04	0.5	0.01
Symptoms	0.8	0.001	0.8	0.001	0.7	0.001	0.4	0.006	0.4	0.05	0.7	0.001
ADL	0.4	0.002	0.4	0.07	0.6	0.001	0.3	0.03	0.2	0.32	0.6	0.001
Sport/Rec	0.4	0.003	0.5	0.01	0.6	0.001	0.3	0.05	0.1	0.65	0.6	0.001
QOL	0.4	0.005	0.5	0.01	0.6	0.002	0.2	0.12	0.1	0.58	0.6	0.002

The association between KOOS subscale scores and the relative difference in muscle strength for knee flexion showed significant and moderate to high correlation (0.5–0.7) for all external ring fixation group subscales. Moderate to low correlations (0.1–0.4) were observed for the intramedullary nailing group (Table 4).

## Discussion

Regardless of the surgical method, i.e., external ring fixation or intramedullary nailing, muscle strength of knee flexion and extension was still markedly decreased one year after sustaining a tibial shaft fracture. We observed no statistically significant difference in recovery of muscle strength between treatment with intramedullary nailing and external ring fixation. Results indicate that a decrease in muscle strength of the injured leg is associated with worse patient-reported outcomes for all five KOOS subscales.

### Intramedullary nailing versus external ring fixation

To the authors' knowledge, this study is the first to report prospective data on the recovery of knee flexion and knee extension strength between intramedullary nailing and external ring fixation for treating tibial shaft fractures. Results showed no significant difference in recovery of muscle strength between treatment with intramedullary nailing or external ring fixation. The relative difference a one-year between intramedullary nailing and external ring fixation was below 8% (knee extension and knee flexion), indicating that other factors than the two surgical procedures may influence the prolonged recovery of muscle strength.

Prior to the present study, we speculated that external ring fixation may be superior in the recovery of muscle strength due to the minimally invasive procedure compared to intramedullary nailing necessitating invasive surgery through the knee joint. However, both surgical treatment options offer free walking support for patients following surgery. Due to pain during the first weeks after surgery, differences in physical activity and the associated loss of muscle strength due to inactivity between patients treated with intramedullary nailing or external ring fixation may be limited.

A common complication of intramedullary nailing is anterior knee pain, which affects about 23% of patients [10]. The association between anterior knee pain and decreased muscle strength has also been established.[19, 20] Pain reporting of patients from the present study is available from the IMVEX trial, showing low pain scores (resting pain VAS < 0.9 and worst pain during the last 24 h VAS < 2.9) for both the intramedullary nailing and the external ring fixation group, and no significant difference between groups [26].

Previous reports on the recovery in muscle strength following tibial shaft fractures are based on intramedullary nailing with an infrapatellar approach [6, 7, 17, 19, 20]. In recent years, an alternative surgical approach using suprapatellar access to the knee for nail insertion has gained popularity and become more widespread [32]. This method was introduced at the hospitals participating in the IMVEX trial around the time when inclusion was initiated. Given the pragmatic design of the IMVEX trail, the choice between supra- and infrapatellar approach for nail insertion was left at the surgeons’ discretion [23]. Several recent studies have reported less pain and superior function in patients operated on with the suprapatellar approach compared to the traditional infrapatellar approach [33–35]. Seventy-five per cent of patients in the intramedullary nailing group in the present study were treated by a suprapatellar approach. The reduction of anterior knee pain associated with the suprapatellar approach may partially explain the comparable muscle strength between intramedullary nailing and external ring fixation.

### Injured versus non-injured leg

At one-year follow-up, the knee flexion and knee extension strength of the injured leg was about 85% compared to the non-injured leg (knee extension: 15.4% (95%CI 10.0–20.8), knee flexion 14.7% (95%CI 9.5–19.9)—results indicating that regaining of normal muscle strength following a tibial shaft fracture takes considerable time. Few studies have previously analysed the recovery of muscle strength following tibial shaft fractures [6, 7, 17–20]. Comparable to the present study, Larsen et al. [6] and Gaston et al. [17] reported 75% to 85% of normal muscle strength in the lower extremities one year after a tibial shaft fracture. Vaistö et al. [19] and Larsen et al. [7] examined the recovery in muscle strength for knee extension and flexion in the long-term (5 to 8 years after fracture) and reported almost balanced muscle strength between legs, supporting that a prolonged recovery period is needed to reach pre-fractured muscle strength following a tibial shaft fracture.

### Associations between KOOS subscale scores and relative difference in muscle strength

One of the most important findings from the present study is the moderate to high correlations observed between KOOS subscale scores and the relative difference in muscle strength for knee extension and knee flexion. Results indicate that a difference in muscle strength between the injured and non-injured leg is associated with worse patient-reported outcomes for all five KOOS subscales. These results are evident regardless of treatment with intramedullary nailing or external ring fixation. Results may suggest the need for focus on knee extension and knee flexion muscle strength during rehabilitation following tibial shaft fractures. Intervention studies are needed to investigate whether including focused muscle strength training can improve patient-reported outcomes following tibial shaft fractures.

The existing literature included little information regarding the association between muscle strength and patient-reported outcomes. Comparable to the result of the present study, Larsen et al. [6] reported a moderate correlation between the decrease in muscle strength and the generic patient-reported score Eq. 5d. In contrast, the present study used the body-region-specific KOOS score to analyse the association with muscle strength. The KOOS may be more sensitive in capturing knee-related complaints than the Eq. 5d score, which may explain the higher correlation observed in the present study for most analyses. Comparable results are also reported by Larsen et al. [36] when nailing a femoral shaft fracture.

The low to moderate correlations between KOOS subscale scores and flexion strength in the nailing group are surprising, especially compared to the moderate to high correlation found in the external ring fixation group. This might represent a difference between groups in the ability to activate the flexion muscles in a functional setting regardless of strength.

### Limitations

This explorative reporting of the IMVEX trial was not powered to analyse muscle strength used for the present study. Furthermore, data from muscle strength was only available from 53 of the included 67 patients from the IMVEX trial, given the possible difference between the two treatment groups in baseline characteristics. Finally, other studies have reported differences in development in muscle strength following tibial fractures between age groups, trauma injury and open/closed fracture. This was not considered for the analyses due to the limited sample size [17].

This is an explorative report from the IMVEX trial; therefore, results and related clinical implications should be interpreted cautiously.

## Conclusion

Regardless of the surgical method, i.e., external ring fixation or intramedullary nailing, muscle strength of knee flexion and extension was still markedly decreased one year after sustaining a tibial shaft fracture. We observed no statistically significant difference in recovery of muscle strength between treatment with intramedullary nailing and external ring fixation. Results indicate that a decrease in muscle strength of the injured leg is associated with worse patient-reported outcomes for all five KOOS subscales.

## Data Availability

No datasets were generated or analysed during the current study.

## References

[1] Larsen P, Elsoe R, Hansen SHSH, Graven-Nielsen T, Laessoe U, Rasmussen S (2015) Incidence and epidemiology of tibial shaft fractures. Injury 46(4):746–750 25636535 10.1016/j.injury.2014.12.027

[2] Bhandari M, Guyatt G, Tornetta P III et al (2008) Study to prospectively evaluate reamed intramedually nails in patients with tibial fractures (S.P.R.I.N.T.): study rationale and design. BMC Musculoskelet Disord 9:91 18573205 10.1186/1471-2474-9-91 PMC2446397

[3] Habernek H, Kwasny O, Schmid L, Ortner F (1992) Complications of interlocking nailing for lower leg fractures: a 3-year follow up of 102 cases. J Trauma 33(6):863–869 1474629 10.1097/00005373-199212000-00012

[4] Lefaivre KA, Guy P, Chan H, Blachut PA (2008) Long-term follow-up of tibial shaft fractures treated with intramedullary nailing. J Orthop Trauma 22(8):525–529 18758282 10.1097/BOT.0b013e318180e646

[5] Larsen P, Elsoe R, Graven-Nielsen T, Laessoe U, Rasmussen S (2016) Local and widespread hyperalgesia after isolated tibial shaft fractures treated with intramedullary nailing. Pain Med (Malden, MA) 17(6):1174–1180 10.1093/pm/pnv016 26814252

[6] Larsen P, Elsoe R, Laessoe U, Graven-Nielsen T, Eriksen CB, Rasmussen S (2016) Decreased QOL and muscle strength are persistent 1 year after intramedullary nailing of a tibial shaft fracture: a prospective 1-year follow-up cohort study. Arch Orthop Trauma Surg 136(10):1395–1402 27498104 10.1007/s00402-016-2537-2

[7] Larsen P, Eriksen CB, Stokholm R, Elsoe R (2021) Results following prolonged recovery show satisfactory functional and patient-reported outcome after intramedullary nailing of a tibial shaft fracture: a prospective 5-year follow-up cohort study. Arch Orthop Trauma Surg 141(8):1303–1310 32951059 10.1007/s00402-020-03608-y

[8] Larsen P, Laessoe U, Rasmussen S, Graven-Nielsen T, Berre Eriksen C, Elsoe R (2017) Asymmetry in gait pattern following tibial shaft fractures—a prospective one-year follow-up study of 49 patients. Gait Posture 51:47–51 27701034 10.1016/j.gaitpost.2016.09.027

[9] Sprague S, Heels-Ansdell D, Bzovsky S et al (2021) Prognostic factors for predicting health-related quality of life after intramedullary nailing of tibial fractures: a randomized controlled trial. Bone Jt Open 2(1):22–32 33537673 10.1302/2633-1462.21.BJO-2020-0150.R1 PMC7842162

[10] Hendrickx LAM, Virgin J, van den Bekerom MPJ, Doornberg JN, Kerkhoffs G, Jaarsma RL (2020) Complications and subsequent surgery after intra-medullary nailing for tibial shaft fractures: review of 8110 patients. Injury 51(7):1647–1654 32360087 10.1016/j.injury.2020.04.021

[11] Pham HM, Nguyen SC, Ho-Le TP, Center JR, Eisman JA, Nguyen TV (2017) Association of muscle weakness with post-fracture mortality in older men and women: a 25-year prospective study. J Bone Miner Res 32(4):698–707 27862286 10.1002/jbmr.3037

[12] Brightwell BD, Van Wyngaarden JJ, Samaan MA, Matuszewski PE, Jacobs CA, Noehren B (2023) Factors associated with long-term quadriceps muscle function after surgical fixation of lower extremity fractures. Phys Ther 103(10):pzad108 37581587 10.1093/ptj/pzad108 PMC11009693

[13] Liu CJ (2009) Latham NK (2009) Progressive resistance strength training for improving physical function in older adults. Cochrane Database Syst Rev 3:CD002759 10.1002/14651858.CD002759.pub2 PMC4324332 19588334

[14] Ericsson YB, Roos EM, Dahlberg L (2006) Muscle strength, functional performance, and self-reported outcomes four years after arthroscopic partial meniscectomy in middle-aged patients. Arthritis Rheum 55(6):946–952 17139641 10.1002/art.22346

[15] Mizner RL, Petterson SC, Snyder-Mackler L (2005) Quadriceps strength and the time course of functional recovery after total knee arthroplasty. J Orthop Sports Phys Ther 35(7):424–436 16108583 10.2519/jospt.2005.35.7.424

[16] Szulc P (2020) Impact of bone fracture on muscle strength and physical performance-narrative review. Curr Osteoporos Rep 18(6):633–645 33030682 10.1007/s11914-020-00623-1

[17] Gaston P, Will E, McQueen MM, Elton RA, Court-Brown CM (2000) Analysis of muscle function in the lower limb after fracture of the diaphysis of the tibia in adults. J Bone Jt Surg 82(3):326–331 10.1302/0301-620x.82b3.9806 10813163

[18] Nyland J, Bealle DP, Kaufer H, Johnson DL (2001) Long-term quadriceps femoris functional deficits following intramedullary nailing of isolated tibial fractures. Int Orthop 24(6):342–346 11294427 10.1007/s002640000191 PMC3619922

[19] Vaisto O, Toivanen J, Kannus P, Jarvinen M (2007) Anterior knee pain and thigh muscle strength after intramedullary nailing of a tibial shaft fracture: an 8-year follow-up of 28 consecutive cases. J Orthop Trauma 21(3):165–171 17473752 10.1097/BOT.0b013e31803773cd

[20] Vaisto O, Toivanen J, Kannus P, Jarvinen M (2004) Anterior knee pain and thigh muscle strength after intramedullary nailing of tibial shaft fractures: a report of 40 consecutive cases. J Orthop Trauma 18(1):18–23 14676552 10.1097/00005131-200401000-00004

[21] Canseco K, Becker BM, Muscott RK, Schmeling GJ, Fritz JM (2024) Gait and strength assessment following surgical repair by intramedullary nailing of isolated tibial shaft fracture. J Orthop Res 42(3):618–627 37804214 10.1002/jor.25704

[22] Toivanen JA, Vaisto O, Kannus P, Latvala K, Honkonen SE, Jarvinen MJ (2002) Anterior knee pain after intramedullary nailing of fractures of the tibial shaft. A prospective, randomized study comparing two different nail-insertion techniques. J Bone Jt Surg 84-A(4):580–585 10.2106/00004623-200204000-00011 11940618

[23] Stokholm R, Larsen P, Rölfing JD et al (2023) Intramedullary nailing versus external ring fixator for treatment of tibial fractures—a study protocol for a randomised clinical trial. Dan Med J 71(1):A05230281 38235983

[24] Frihagen F, Madsen JE, Sundfeldt M et al (2020) Taylor spatial frame or reamed intramedullary nailing for closed fractures of the tibial shaft: a randomized controlled trial. J Orthop Trauma 34(11):612–619 33065663 10.1097/BOT.0000000000001802

[25] Ramos T, Eriksson BI, Karlsson J et al (2014) Ilizarov external fixation or locked intramedullary nailing in diaphyseal tibial fractures: a randomized, prospective study of 58 consecutive patients. Arch Orthop Trauma Surg 134(6):793–802 24664228 10.1007/s00402-014-1970-3

[26] Stokholm R, Larsen P, Rölfing JD et al (2025) Intramedullary nailing versus external ring fixation for the treatment of tibial shaft fractures: a multicentre randomized controlled clinical trial (IMVEX). Bone Jt J 107-B:353–361 10.1302/0301-620X.107B3.BJJ-2024-0812.R1 40020724

[27] Butcher NJ, Monsour A, Mew EJ et al (2022) Guidelines for reporting outcomes in trial reports: the CONSORT-outcomes 2022 extension. JAMA 328(22):2252–2264 36511921 10.1001/jama.2022.21022

[28] Rathleff CR, Baird WN, Olesen JL, Roos EM, Rasmussen S, Rathleff MS (2013) Hip and knee strength is not affected in 12–16 year old adolescents with patellofemoral pain—a cross-sectional population-based study. PLoS ONE 8(11):e79153–e79153 24236101 10.1371/journal.pone.0079153 PMC3827322

[29] Roos EM, Roos HP, Lohmander LS, Ekdahl C, Beynnon BD (1998) Knee Injury and osteoarthritis outcome score (KOOS)—development of a self-administered outcome measure. J Orthop Sports Phys Ther 28(2):88–96 9699158 10.2519/jospt.1998.28.2.88

[30] Erin-Madsen N, Aasvang TK, Viberg B, Bloch T, Brix M, Tengberg PT (2019) Knee pain and associated complications after intramedullary nailing of tibial shaft fracture. Dan Med J 66(8):A5554 31315794

[31] Schober P, Boer C, Schwarte LA (2018) Correlation coefficients: appropriate use and interpretation. Anesth Analg 126(5):1763–1768 29481436 10.1213/ANE.0000000000002864

[32] Ringenberg JD, Tobey JL, Horinek JL, Teague DC (2022) Suprapatellar versus infrapatellar approach for intramedullary nail fixation of tibial shaft fractures: a review of the literature. OTA Int 5(1):e196 35187413 10.1097/OI9.0000000000000196 PMC8843371

[33] Yang L, Sun Y, Li G (2018) Comparison of suprapatellar and infrapatellar intramedullary nailing for tibial shaft fractures: a systematic review and meta-analysis. J Orthop Surg Res 13(1):146 29898758 10.1186/s13018-018-0846-6 PMC6001044

[34] Sepehri A, You D, Lobo AA, Schneider P, Lefaivre KA, Guy P (2022) Comparison of patient-reported outcomes after suprapatellar versus infrapatellar nailing techniques for tibial shaft fractures: a systematic review and meta-analysis. J Orthop Trauma 36(6):e208–e214 34799545 10.1097/BOT.0000000000002303

[35] Al-Azzawi M, Davenport D, Shah Z, Khakha R, Afsharpad A (2021) Suprapatellar versus infrapatellar nailing for tibial shaft fractures: a comparison of surgical and clinical outcomes between two approaches. J Clin Orthop Trauma 17:1–4 33717965 10.1016/j.jcot.2021.01.009 PMC7920150

[36] Larsen P, Elsoe R, Graven-Nielsen T, Laessoe U, Rasmussen S (2014) Decreased muscle strength is associated with impaired long-term functional outcome after intramedullary nailing of femoral shaft fracture. Eur J Trauma Emerg Surg 41(6):673–681 26038009 10.1007/s00068-014-0488-2

